# Draft Genome Sequence of *Clostridium* sp. Strain E02, Isolated from an Estuarine Environment

**DOI:** 10.1128/MRA.01579-18

**Published:** 2019-01-10

**Authors:** Michael Christopher Macey, Elliot Curtis-Harper, Karen Olsson-Francis

**Affiliations:** a School of Environment, Earth and Ecosystem Sciences, Faculty of Science, Technology, Engineering and Mathematics, The Open University, Milton Keynes, United Kingdom; b School of Physical Sciences, Faculty of Science, Technology, Engineering and Mathematics, The Open University, Milton Keynes, United Kingdom; Louisiana State University

## Abstract

Here, we report the draft genome sequence of a strain of Clostridium isolated from sediment collected from an estuarine environment. The strain was isolated using a minimal medium designed to select for chemoautotrophic microorganisms. The strain may represent a novel species within the genus Clostridium, and this genome sequence enables further investigation into the genetic and metabolic diversity of this organism.

## Abstract (precis)

Here, we report the draft genome sequence of a strain of Clostridium isolated from sediment collected from an estuarine environment. The strain was isolated using a minimal medium designed to select for chemoautotrophic microorganisms.

## ANNOUNCEMENT

Anoxic sediments in estuarine systems are proposed as analogue sites to investigate the habitability of the ancient lake system at Gale Crater on Mars because of similarities in temperature, salinity, redox, and pH regimes (1–5). Characterization of microbes at these sites allows for hypothesis development with regard to potential viable Martian metabolisms. Clostridium sp. strain E02 was isolated from sediment from the River Dee, which flows into the Liverpool Bay, in the United Kingdom (53°21′15.40″ N, 3°10′24.95″ W). The isolation involved enrichment of sediment in a minimal growth medium (5). Clostridium sp. strain E02 is a rod-shaped, anaerobic, Gram-positive bacterium belonging to the family Clostridiaceae in the order Clostridiales (5).

The strain was cultured in anaerobic lysogeny broth (LB) at 25°C prior to DNA extraction using the Griffiths technique (6). Genome sequencing was performed by MicrobesNG (https://microbesng.uk/). DNA quantification and library preparation were carried out on a Hamilton Microlab Star automated liquid handling system. Libraries were quantified using the Kapa Biosystems library quantification kit for Illumina on a Roche light cycler 96 quantitative PCR (qPCR) machine. Libraries were sequenced on an Illumina HiSeq instrument, using a 250-bp paired-end protocol. A total of 553,783 trimmed reads were produced using Trimmomatic (v0.30) with a sliding window quality cutoff of Q15. *De novo* assembly was performed using SPAdes (v3.7; default settings) (7, 8). Coverage of 60× was achieved during sequencing, calculated using BWA, SAMtools (v0.1.19), and BEDTools genomecov (v2.2.7), all with default settings (9–11). Annotation was performed using the Rapid Annotations using Subsystems Technology (RAST) annotation server (v2.0) with the classic RAST pipeline (12). The presence of genes involved in nitrogen and sulfur cycling was investigated to assess the potential for growth in oligotrophic conditions. Gene screening was performed with BlastKOALA (v2.1; default settings) (13) and supported with BLAST searches against the genome sequence using BioEdit (v7.0.5; default settings) (14).

The draft genome of Clostridium sp. strain E02 is 4.08 Mb in size, with a G+C content of 40% and an *N*_50_ value of 146,559 bp. The genome is composed of 114 contigs, including 3,850 coding sequences, one 16S rRNA gene copy, and 68 tRNAs. Analysis of the 16S rRNA gene using the SILVA alignment, classification and tree service (15) identified Clostridium sp. strain E02 as closely related to Clostridium saccharolyticum WM1, with 98% sequence identity. The genomes of both strains were compared using the genome to genome distance calculator (GGDC) (v2.1; default settings) (16) and the average nucleotide identity (ANI) calculator (default settings) (17). GGDC (23.1%) and ANI (76.5%) scores support that Clostridium sp. strain E02 is genetically distinct from the most related species. The genome was shown to contain genes encoding a molybdenum-iron nitrogenase (*nifDKH*), an assimilatory sulfate reductase (*cysND*), and an adenylsulfate reductase (*aprAB*).

### Data availability.

This whole-genome shotgun project has been deposited at DDBJ/ENA/GenBank under the accession number RJLQ00000000. The version described in this paper is version RJLQ01000000. The strain is available from the authors upon request. Raw sequencing reads for Clostridium sp. strain E02 are available in the NCBI Sequence Read Archive under accession number SRR8246104.
